# *XRCC1*和*XRCC3*单核苷酸多态性与晚期非小细胞肺癌铂类药物化疗疗效的相关性

**DOI:** 10.3779/j.issn.1009-3419.2011.12.03

**Published:** 2011-12-20

**Authors:** 崇安 许, 小杰 王, 晔 张, 琳 李

**Affiliations:** 1 110032 沈阳，中国医科大学附属第四医院肿瘤内科 Department of Medical Oncology, the Fourth Affiliated Hospital, China Medical University, Shenyang 110032, China; 2 110001 沈阳，中国医科大学附属第一医院肿瘤内科 Department of Medical Oncology, the First Affiliated Hospital, China Medical University, Shenyang 110001, China

**Keywords:** 肺肿瘤, XRCC1, XRCC3, 单核苷酸多态性, 化疗敏感性, Lung neoplasms, X-ray repair cross complementing protein 1 (XRCC1), X-ray repair cross complementing protein 3 (XRCC3), Single nucleotick polymorphism, Chemotherapy sensitivity

## Abstract

**背景与目的:**

DNA修复基因多态性预测铂类药物化疗敏感性对非小细胞肺癌(non-small cell lung cancer, NSCLC)个体化治疗具有重要意义。本研究旨在探讨X线修复交错互补基因1(X-ray repair cross complementing gene 1, XRCC1)和X线修复交错互补基因3(X-ray repair cross complementing gene 3, XRCC3)单核苷酸多态性与晚期NSCLC患者对铂类药物化疗疗效的关系。

**方法:**

采用PCR-RFLP方法检测130例以含铂方案化疗的晚期NSCLC患者外周血DNA中XRCC1 Arg194 Trp、Arg399 Gln和XRCC3 Thr241 Met基因多态性, 分析其基因型与化疗疗效的关系。

**结果:**

130例晚期NSCLC患者采用含铂方案化疗2个周期后, 化疗总有效率为33.8%。XRCC1 194和399基因多态性与铂类药物化疗敏感性相关, 而XRCC3 241基因多态性与化疗敏感性无关(*P*=0.145)。携带至少1个XRCC1 194 Trp等位基因者化疗有效率至少是携带Arg/Arg基因型患者的2.5倍(42.1% *vs* 22.2%, OR=2.545, 95%CI:1.159-5.590, *P*=0.020)。携带XRCC1 399 Arg/Arg基因型者的化疗有效率为45.5%, 明显高于携带至少1个Gln等位基因者(21.9%)(OR=0.336, 95%CI:0.156-0.722, *P*=0.005)。XRCC1 194和399基因多态性之间存在联合作用, 同时携带至少1个XRCC1 194 Trp等位基因和399 Arg/Arg基因型者的化疗有效率明显高于同时携带194 Arg/Arg和399 Arg/Gln基因型者(44.4% *vs* 18.8%, OR=3.467, 95%CI:1.223-9.782, *P*=0.019)。*XRCC1*和*XRCC3*基因多态性在化疗敏感性方面存在一定的联合作用, 携带至少1个XRCC1 194 Trp等位基因和399 Arg/Arg野生型基因同时又携带XRCC3 241 Thr/Met基因型者的化疗有效率明显高于其它基因型携带者。

**结论:**

XRCC1和XRCC3的多态联合可能与晚期NSCLC患者对铂类药物化疗疗效具有相关性。

## Abstract (en)

**Background and objective:**

DNA repair gene polymorphisms can be used to predict the sensitivity of platinum-based chemotherapy.Thus, such polymorphisms are important for the individual treatment of non-small cell lung cancer (NSCLC).The aim of this study is to investigate the relationship between X-ray repair cross complementing protein 1 (XRCC1) and X-ray repair cross complementing protein 3 (*XRCC3*) gene polymorphisms and the chemosensitivity of platinum-based chemotherapy in patients with advanced NSCLC.

**Methods:**

Genomic DNA were extracted from the sera of a total of 130 patients with advanced NSCLC who received platinum-based chemotherapy.XRCC1 Arg194 Trp, Arg399 Gln, and XRCC3 Thr241 Met were genotyped using the polymerase chain reaction-restriction fragment length polymorphism method, and the relationship between *XRCC1* and *XRCC3* polymorphisms and chemotherapy sensitivity was analyzed.

**Results:**

A total of 130 patients with advanced NSCLC received platinum-based chemotherapy, with an overall response rate of 33.8% after two chemotherapy cycles.The XRCC1 194 and 399 genetic polymorphisms, but not XRCC3 241, were found to be related to the chemosensitivity.The objective response rate of the patients with at least one XRCC1 194 Trp allele was 2.5 times higher than that of Arg/Arg genotype carriers (42.1% *vs* 22.2%, OR=2.545, 95%CI:1.159-5.590, *P*=0.020).The objective response rate of the XRCC1 399 Arg/Arg genotype carriers was significantly higher than that of the patients with at least one Gln allele (45.5% *vs* 21.9%, OR=0.336, 95%CI:0.156-0.722, *P*=0.005).Combined effects between XRCC1 194 and XRCC1 399 were observed.The objective response rate of the patients with at least one XRCC1 194 Trp allele and a 399 Arg/Arg genotype was significantly higher than that of patients with 194 Arg/Arg and 399 Arg/Gln genotypes (44.4% *vs* 18.8%, OR=3.467, 95%CI:1.223-9.782, *P*=0.019).Moreover, XRCC1 and XRCC3 have a combined effect in predicting chemosensitivity.Patients with XRCC3 241 Thr/Met, 399 Arg/Arg, and at least one XRCC1 194 Trp allele simultaneously showed an improved objective response rate.

**Conclusion:**

The combination of the *XRCC1* and *XRCC3* polymorphisms may be associated with patient sensitivity to platinum-based chemotherapy in advanced NSCLC.

铂类药物(顺铂或卡铂)是治疗非小细胞肺癌(non-small cell lung cancer, NSCLC)的重要药物, 其与长春瑞滨、吉西他滨、紫杉醇、多西他赛任一联合的两药方案对NSCLC患者化疗的有效率相似^[1]^, 与非铂类方案相比, 含铂两药方案化疗能够延长NSCLC患者的生存期, 并提高其生活质量^[2]^, 含铂两药方案现已成为治疗进展期NSCLC的标准一线方案。铂类药物主要通过损伤DNA而导致细胞死亡^[3]^, 而机体的DNA损伤修复系统可以修复这类损伤, 从而影响铂类药物化疗的敏感性。由于个体之间对DNA损伤后的修复能力有很大的差异^[4, 5]^, 因此, 常可导致即使同样是NSCLC, 甚至是病理类型和临床分期相同的患者采用相同的治疗方案, 其治疗效果和不良反应却明显不同。DNA修复基因的过表达及DNA修复基因单核苷酸多态性(single nucleotide polymorphism, SNP)可改变DNA修复能力, 因此, 检测DNA修复基因的SNP可以预测个体肿瘤化疗的敏感性和预后。

X线修复交错互补基因1(X-ray repair cross complementing gene 1, XRCC1)是DNA碱基切除修复和单链断裂修复系统中的重要成分, 参与铂类药物等引起的DNA修复过程, 该基因编码区存在导致氨基酸取代的SNP, 这些SNP影响XRCC1的活性^[6, 7]^。虽然许多功能性研究已报告了XRCC1对肿瘤的影响, 但其SNP与NSCLC铂类药物化疗敏感性关联的研究结果却不尽相同。X线修复交错互补基因3(X-ray repair cross complementing gene 3, XRCC3)是Rad51 DNA修复基因家族成员, 虽然在DNA双链断裂同源性重组修复中扮演重要角色^[8]^, 但其Thr241Met位点的SNP与铂类药物化疗敏感性的关系尚不清楚。本研究报告130例晚期NSCLC患者XRCC1和XRCC3的SNP以及它们与铂类药物化疗疗效的关系, 为个体化化疗提供依据。

## 1 材料与方法

### 1.1 研究对象

2008年8月-2010年3月中国医科大学附属第四医院收治的经病理证实的初治晚期NSCLC患者130例, 其中男性90例, 女性40例, 均为汉族; 中位年龄62(28-83)岁; 吸烟80例, 不吸烟50例; 腺癌76例, 鳞癌46例, 其它类型8例; 临床分期:Ⅲb期58例, Ⅳ期72例; ECOG PS评分:0分44例, 1分86例。所有患者均有可测量病灶并均接受了2个-6个周期(平均4个周期)含铂两药方案化疗。具体化疗方案如下:长春瑞滨+铂类(顺铂或卡铂)82例, 吉西他滨+铂类26例, 紫杉醇类(紫杉醇或多西他赛)+铂类22例。

### 1.2 样本收集

血液标本在患者知情同意的情况下, 于化疗前抽取静脉血5 mL, 静置后取血凝块并提取DNA, 将提取的DNA置于-20 ℃低温冰箱中备用。

### 1.3 疗效评定

所有患者均经含铂两药方案化疗2个周期后根据RECIST标准评定疗效, 分为完全缓解(complete response, CR)、部分缓解(partial response, PR)、疾病稳定(stable disease, SD)和疾病进展(progressive disease, PD)。客观缓解=CR+PR, 未缓解=SD+PD。

### 1.4 基因型分析

采用PCR-RFLP方法进行XRCC1、XRCC3基因型检测。引物序列及扩增片段大小见表 1。25 μL PCR反应混合液中含1 μL DNA、0.4 μmol/L引物各1 μL、0.1 mmol/L dNTP 2 μL、Taq聚合酶0.125 U、5×反应缓冲液5 μL。PCR反应条件:95 ℃预变性5 min; 95 ℃ 45 s, 61 ℃ 45 s, 72 ℃ 45 s, 35个循环; 72 ℃延伸10 min。分别取5 μL PCR产物与相应的核酸内切酶37 ℃温育过夜, XRCC1 Arg194 Trp、Arg399 Gln及XRCC3 Thr241 Met核酸内切酶分别为*Pvu*Ⅱ、*Nci*Ⅰ和*Nla*Ⅲ。3.0%琼脂糖凝胶电泳分析酶切产物。随机抽取30%样本, 双盲法重复检测加以验证, 结果完全一致。

**1:** PCR引物及扩增片段大小 PCR primers and product sizes

Gene polymoyphism (length)	Primer sequence
XRCC1 code 194 (485 bp)	5′-GCCAGGGCCCCTCCTTCAA-3′
	5′-TACCCTCAGACCCACGAGT-3′
XRCC1 code 399 (384 bp, 133 bp)	5′-TCCTCCACCTTGTGCTTTCT-3′
	5′- AGTAGTCTGCTGGCTCTGGG -3′
XRCC3 code 241 (315 bp, 141 bp)	5′-GGTCGAGTGACAGTCCAAAC -3′
	5′-TGCAACGGCTGAGGGTCTT -3′

### 1.5 统计分析

应用SPSS 17.0进行统计学分析, 采用*χ*^2^检验或*Fisher’s*精确概率法比较不同基因型之间化疗疗效的差异, 采用非条件*Logistic*回归分析计算优势比(odds ratio, OR)及其95%可信区间(confidence interval, CI)评价不同基因型及多态位点间联合与疗效的相关性。所有检验均为双侧, *P* < 0.05为差异有统计学意义。

## 2 结果

### 2.1 疗效分析

130例晚期NSCLC患者经含铂两药方案化疗2个周期后, CR 0例, PR 44例(33.8%), SD 54例(41.6%), PD 32例(24.6%), 化疗总有效率为33.8%(44/130)。

### 2.2 基因分布

130例晚期NSCLC患者, 在XRCC1 Arg194Trp位点中, 携带Arg/Arg、Arg/Trp和Trp/Trp基因型患者分别为54例(41.5%)、40例(30.8%)和36例(27.7%)。在XRCC1 Arg399 Gln位点中, 携带Arg/Arg、Arg/Gln和Gln/Gln基因型患者分别为66例(50.8%)、54例(41.5%)和10例(7.7%)。在XRCC3 Thr241 Met位点中, 携带Thr/Thr和Thr/Met基因型患者分别为114例(87.7%)和16例(12.3%), 未检测到Met/Met纯合突变基因型。

### 2.3 基因型与化疗疗效的关系

从表 2可见, 携带XRCC1 194 Arg/Arg、Arg/Trp和Trp/Trp基因型NSCLC患者的化疗有效率分别为22.2%、35.0%和50.0%, 有明显差异(*χ*^2^=7.478, *P*=0.024)。携带Trp/Trp基因型患者的化疗有效率是Arg/Arg基因型携带者的3.5倍(*P*=0.006)。携带至少1个Trp等位基因(Arg/Trp和Trp/Trp基因型)患者的化疗有效率至少是携带Arg/Arg基因型患者的2.5倍(*P*=0.018)。携带XRCC1 399 Arg/Arg、Arg/Gln和Gln/Gln基因型患者的化疗有效率分别为45.5%、25.9%和0, 携带XRCC1 399 Arg/Arg基因型患者的化疗有效率明显高于其它基因型携带者。携带XRCC3 241 Thr/Thr与Thr/Met基因型患者化疗有效率分别为31.6%和50.0%, 二者无统计学差异(*P*=0.145)。

**2:** XRCC1和XRCC3基因型与NSCLC化疗敏感性的关系 The relationship between XRCC1 and XRCC3 genotypes and chemosensitivity of NSCLC

Genotype	*n* (%)	Response to chemotherapy [n (%)]		*P*	OR	95%CI
		CR+PR	SD+PD			
XRCC1 Arg194 Trp						
Arg/Arg	54 (41.5)	12 (22.2)	42 (77.8)	-	1	-
Arg/Trp	40 (30.8)	14 (35.0)	26 (65.0)	0.171	1.885	0.756-4.696
Trp/Trp	36 (27.7)	18 (50.0)	18 (50.0)	0.006	3.500	1.401-8.744
Arg/Trp+Trp/Trp	76 (58.5)	32 (42.1)	44 (57.9)	0.018	2.545	1.159-5.590
XRCC1 Arg399 Gln						
Arg/Arg	66 (50.8)	30 (45.5)	36 (54.5)	-	1	-
Arg/Gln	54 (41.5)	14 (25.9)	40 (74.1)	0.027	0.420	0.193-0.914
Gln/Gln	10 (7.7)	0 (0)	10 (100.0)	0.005	0	0
Arg/Gln+ Gln/Gln	64 (49.2)	14 (21.9)	50 (78.1)	0.005	0.336	0.156-0.722
XRCC3 Thr241 Met						
Thr/Thr	114 (87.7)	36 (31.6)	78 (68.4)	-	1	-
Thr/Met	16 (12.3)	8 (50.0)	8 (50.0)	0.145	2.167	0.753-6.232
CR:complete response; PR:partial response; SD:stable disease; PD:progressive disease; NSCLC:non-small cell lung cancer.						

年龄、性别、吸烟、病理类型及临床分期与晚期NSCLC患者化疗敏感性均无统计学关联(*P* > 0.05)。

### 2.4 XRCC1 Arg194 Trp和Arg399 Gln多态联合作用与化疗疗效的关系

我们进一步分析了XRCC1 Arg194 Trp和Arg399 Gln两个位点基因型联合与化疗疗效的关系, 结果显示, XRCC1 194和399多态之间在化疗敏感性方面存在明显相互作用, 同时携带XRCC1 194Arg/Arg和399Gln/Gln基因型的10例患者无一例有效, 而携带至少1个XRCC1 194 Trp等位基因同时又携带XRCC1 399 Arg/Arg基因型者的化疗有效率明显高于同时携带194 Arg/Arg和399 Arg/Gln基因型者(表 3)。

**3:** XRCC1 Arg194 Trp和Arg399 Gln多态联合与化疗疗效的关系 Interaction of XRCC1 Arg194 Trp and XRCC1 Arg399 Gln polymorphisms on the chemotherapy response

Genotype		*n* (%)	Response to chemotherapy [n (%)]		*P*	OR	95%CI	*P*
Arg194 Trp	Arg399 Gln		CR+PR	SD+PD				
Arg/Arg	Gln/Gln	10 (7.7)	0 (0)	10 (100.0)	-	-	-	-
Arg/Arg	Arg/Gln	32 (24.6)	6 (18.8)	26 (81.2)	-	1	-	-
Arg/Arg	Arg/Arg	12 (9.2)	6 (50.0)	6 (50.0)	0.059^*^	4.333	1.029-18.257	0.046
Arg/Trp+Trp/Trp	Arg/Arg	54 (41.5)	24 (44.4)	30 (53.6)	0.016	3.467	1.229-9.782	0.019
Arg/Trp+Trp/Trp	Arg/Gln	22 (16.8)	8 (36.4)	14 (63.6)	0.147	2.476	0.715-8.574	0.152
^*^:*Fisher’s* exact test.								

### 2.5 XRCC1与XRCC3联合基因型与化疗疗效的关系

本研究进一步分析了*XRCC1*与*XRCC3*两个基因不同多态基因型联合与化疗敏感性的关系, 发现*XRCC1*与XRCC3多态之间在化疗敏感性方面存在明显的相互作用; 携带至少1个XRCC1 194 Trp等位基因和399 Arg/Arg野生型基因同时又携带XRCC3 241 Thr/Met基因型者的化疗有效率明显高于其它基因型携带者(表 4)。

**4:** XRCC1和XRCC3联合基因型与化疗疗效的关系 Associations of XRCC1 and XRCC3 genotypes with the efficacy of chemotherapy

Combined genotype	*n* (%)	Response to chemothery [n (%)]		OR	95%CI	*P*
		CR+PR (%)	SD+PD (%)			
XRCC1 194 Arg/Arg+399(Arg/Gln+Gln/Gln)+XRCC3 241 Thr/Thr	48 (36.9)	12 (25.0)	36 (75.0)	0.222	0.054-0.923	0.038
XRCC1 194 Arg/Arg+399(Arg/Gln+Gln/Gln)+XRCC3 241 Thr/Met	2 (1.5)	0	2	-	-	-
XRCC1 194 Arg/Arg+399Arg/Arg+XRCC3 241 Thr/Thr	10 (7.8)	4 (40.0)	6 (60.0)	0.444	0.074-2.666	0.374
XRCC1 194 Arg/Arg+399Arg/Arg+XRCC3 241 Thr/Met	2 (1.5)	2	0	-	-	-
XRCC1 194 (Arg/Trp+Trp/Trp)+399 Arg/Arg+XRCC3 241 Thr/Thr	44 (33.8)	18 (40.9)	26 (59.1)	0.462	0.114-1.873	0.279
XRCC1 194 (Arg/Trp+Trp/Trp)+399 Arg/Arg+XRCC3 241 Thr/Met	10 (7.8)	6 (60.0)	4 (40.0)	1	-	-
XRCC1 194 (Arg/Trp+Trp/Trp)+399Arg/Gln+XRCC3 241 Thr/Thr	12 (9.2)	2 (16.7)	10 (83.3)	0.133	0.018-0.962	0.046
XRCC1 194 (Arg/Trp+Trp/Trp)+399Arg/Gln+XRCC3 241 Thr/Met	2 (1.5)	0	2 (100)	-	-	-

## 3 讨论

人类细胞中存在大量DNA修复基因, 它们对某些类型的DNA损伤具有特异性修复能力, 对避免基因突变、维护基因组的稳定性和完整性具有重要的作用。许多DNA修复基因具有SNP, 导致氨基酸替代的SNP可能改变修复酶的活性, 并且可能是导致个体DNA损伤修复能力差异的重要原因^[9]^。研究^[10]^表明, DNA修复能力与肿瘤易感性密切相关, DNA修复能力低肿瘤易感性高; 而在肿瘤治疗过程中, DNA修复能力的增加却阻碍了疗效的发挥, 肿瘤细胞的DNA修复能力与化疗敏感性密切相关。

XRCC1的SNP可影响XRCC1蛋白的正常功能, 从而影响DNA修复能力^[11]^。XRCC1 SNP与铂类药物化疗敏感性相关, 携带XRCC1 194 Trp等位基因的NSCLC患者对铂类药物化疗敏感, 而携带Arg/Arg基因型患者对铂类药物化疗失败的风险几乎是携带至少1个Trp等位基因患者的3倍。携带至少1个XRCC1 399 Gln等位基因的患者对铂类药物化疗失败的风险是399 Arg/Arg携带者的2.7倍。而且XRCC1 194和399这两个位点的SNP存在联合作用, 同时携带194 Arg/Trp和399 Arg/Arg基因型患者对铂类药物化疗的敏感性更高^[12]^。洪成雨等^[13]^研究结果显示, 仅XRCC1 194基因多态与NSCLC患者铂类药物化疗的敏感性相关, 而Arg399 Gln基因多态与化疗敏感性无关, 而且XRCC1 194和399两个位点的SNP也无联合作用。姚成云^[14]^和赵万^[15]^的研究虽然证实了XRCC1 399基因多态与NSCLC患者铂类药物化疗敏感性相关, 但其结果却显示, 携带XRCC1 399 Gln突变基因患者的化疗反应率反而明显高于399 Arg/Arg野生型基因携带者。

本研究结果表明, XRCC1 Arg194 Trp和Arg399 Gln两个位点的SNP均与NSCLC患者铂类药物的化疗敏感性密切相关。携带XRCC1 194 Trp纯合突变基因患者的化疗有效率是Arg/Arg野生型基因携带者的3.5倍(*P*=0.006)。携带至少1个Trp等位基因患者的化疗有效率至少是携带Arg/Arg基因型患者的2.5倍(*P*=0.018)。携带XRCC1 399 Arg/Arg野生型基因患者的化疗有效率明显高于其它基因型携带者。XRCC1 194和399两个位点基因多态之间在化疗敏感性方面存在明显的相互作用, 携带至少1个XRCC1 194 Trp等位基因同时又携带XRCC1 399 Arg/Arg基因型患者的化疗有效率明显高于同时携带194 Arg/Arg和399 Arg/Gln基因型患者。本研究结果表明, 携带XRCC1 194 Trp突变基因的患者对铂类药物化疗敏感, 同时携带XRCC1 194 Trp突变基因和399 Arg/Arg野生型基因的患者对铂类药物化疗更敏感, 而携带XRCC1 194 Arg/Arg野生型基因和399 Gln突变基因的患者对铂类药物化疗耐药。

许多研究报告了XRCC3 241 SNP与肺癌易感性的关联, 但其研究结果却不尽相同, 就目前的研究结果而言, 尚无证据表明XRCC3 241多态性与中国人肺癌易感性相关^[16, 17]^。而XRCC3 Thr241 Met的SNP与铂类药物化疗敏感性关系的研究较少, 国内仅见任胜祥等^[18]^报告了ERCC1和XRCC3 241基因多态性在接受含铂方案化疗NSCLC中的疗效预测作用, 其结果显示, 130例晚期NSCLC患者中85.4%携带XRCC3 241 Thr/Thr基因型, 16.4%携带Thr/Met或Met/Met基因型。XRCC3 241各基因型之间化疗有效率无统计学差异。本研究结果显示, 在130例晚期NSCLC患者中, 携带Thr/Thr和Thr/Met基因型患者分别为87.7%和12.3%, 未检测到Met/Met纯合突变基因型。携带XRCC3 241 Thr/Thr和Thr/Met基因型者之间的化疗有效率无统计学差异。但XRCC1和XRCC3多态在化疗敏感性方面却存在明显的交互作用。

综上所述, 本研究发现, XRCC1 194和399基因单核苷酸多态性均与晚期NSCLC患者铂类药物化疗敏感性密切相关, 而XRCC3 241单核苷酸多态性与铂类药物化疗疗效无关。不仅XRCC1 194和399两个位点的基因多态具有联合作用, 而且*XRCC1*与*XRCC3*两个基因多态在铂类药物化疗敏感性方面亦存在明显的交互作用。由此可见, *XRCC1*和*XRCC3*两个基因的多态性可能对临床化疗药物的选择具有指导意义。但由于本研究样本量较少, 在应用于临床之前尚需扩大样本深入研究DNA碱基切除修复系统乃至整个DNA修复系统与铂类药物敏感性的关系, 为肿瘤个体化治疗提供理论依据。

## References

[1] Fisher MD, D' Orazio A (2000). Phase Ⅱ and Ⅲ trials:comparison of four chemotherapy regimens in advanced non small-cell lung cancer (ECOG 1594). Clin Lung Cancer.

[2] Rajeswaran A, Trojan A, Burnand B (2008). Efficacy and side effects of cisplatin and carboplatin-based doublet chemotherapeutic regimens versus non-platinum-based doublet chemotherapeutic regimens as first line treatment of metastatic non-small cell lung carcinoma:a systematic review of randomized controlled trials. Lung Cancer.

[3] Hildebrandt MA, Gu J, Wu X (2009). Pharmacogenomics of platinum-based chemotherapy in NSCLC. Expert Opin Drug Metab Toxicol.

[4] Nisato RE, Tille JC, Pepper MS (2003). Lymphangiogenesis and tumor metastasis. Thromb Haemost.

[5] Kiyohara C, Takayama K, Nakanishi Y (2006). Association of genetic polymorphisms in the base excision repair pathway with lung cancer risk:a meta-analysis. Lung Cancer.

[6] Gurubhagavatula S, Liu G, Park S (2007). *XPD* and *XRCC1* genetic polymorphisms are prognostic factors in advanced non-small-cell lung cancer patients treated with platinum chemotherapy. J Clin Oncol.

[7] Luun RM, Langlois RG, Hsieh LL (1999). *XRCC1* polymorphisms effects on aflatoxin B1-DNA adducts and glycophorin a variant frequency. Cancer Res.

[8] Rosell R, Lord RV, Taron M (2002). DNA repair and cisplatin resistance in non-small-cell lung cancer. Lung Cancer.

[9] Butkiewicz D, Rusin M, Enewold L (2001). Genetic polymorphisms in DNA repair genes and risk of lung cancer. Carcinogenesis.

[10] Wei Q, Frazier ML, Levin B (2000). DNA repair:a double-edged sword. J Natl Cancer Inst.

[11] Shen MR, Jones M, Mohrenweiser H (1998). Nonconservative amino acid substitution variants exist at polymorphic frequency in DNA repair genes in healthy humans. Cancer Res.

[12] Wang ZH, Miao XP, Tan W (2004). Single nucleotide polymorphisms in *XRCC1* and clinical response to platin-based chemotherapy in advanced non-small cell lung cancer. Chin J Cancer.

[b13] 13Hong CY, XU Q, Yue Y, et al. Correlation of the sensitivity of NP chemotherapy in non-small lung cancer with DNA repair gene *XRCC1* polymorphism. 2009, 14(12): 1291-1297.http://www.wanfangdata.com.cn/details/detail.do?_type=perio&id=ez200912011洪成雨, 徐倩, 袁媛, 等. 非小细胞肺癌NP方案化疗敏感性与DNA修复基因*XRCC1*多态性的关系. 癌症, 2009, 14(12): 1291-1297.

[b14] 14Yao CY, Huang XE, Li C, et al. Relationship between the combination of *XRCC1* and *XPD* gene polymorphisms with platinum-based chemotherapy in advanced non-small cell lung cancer. 2010, 30(6): 391-395.http://kns.cnki.net/KCMS/detail/detail.aspx?filename=xzyx201006017&dbname=CJFD&dbcode=CJFQ姚成云, 黄新恩, 黎超, 等. 在晚期非小细胞肺癌中*XRCC1*和*XPD*基因多态性的联合和铂类化疗的关系. 徐州医学院学报, 2010, 30(6): 391-396.

[15] Zhao W, Hu LM, Wang C (2011). Relationship of *XRCC1*, *PARP1* and *APE1* polymorphisms with efficacy of platinum-based chemotherapy for patients with advanced non-small cell lung cancer. Acta universitatis medicinalis nanjing (Natural Science).

[16] Zhang ZL, Zhou CC, Zhang J (2007). Relationship between polymorphisms of DNA repair gene *XRCC3* and susceptibility to lung cancer. Chin J Tuberc Respir Dis.

[17] Hang M, Cai L (2010). *XRCC3* polymorphisms associated with lung cancer:a *meta* analysis. Fujian Med Univ.

[18] Ren SX, Zhou CC, Zhou SW (2009). Predictive role of *ERCC1* and *XRCC3* gene polymorphism on response of platinum-based chemotherapy in advanced NSCLC. J Oncol.

